# Annotations

**Published:** 1907-12-14

**Authors:** 


### Annotations.

### Glasgow University Club, London.

The usual semi-annual dinner of this Club was given in the Trocadero Restaurant on December 6, when the chair was taken by Professor John Ferguson, M.A., LL.D., F.S.A. There was a large attendance of the members and their friends, and among others the Chairman was supported by Sir Wm. Ramsay, K.C.B., Otto Hehner, Esq., F.I.C., Professors D'Arcy Thompson, J. Norman Collie, John M. Thomson, W. P. Ker, and Herbert Jackson, and by Lieutenant-Colonel Leishman, Dr. George A. Heron, Dr. Seton Orr, and Dr. Chalmers Mitchell. In an interesting speech, in proposing "The University and the Club," Professor Ferguson carried his hearers back to the early 'seventies, when he succeeded to the Chair of Chemistry in the University, and when the old College in High Street was the centre of the academic organisation. He compared the state of matters then existing with the present condition of the University, and emphasised particularly the recent developments at Gilmorehill, where large modern laboratories have been erected in connection with the work of each of the several subjects in the faculties of science and medicine. While necessarily interested more directly in the prosecution and extension of scientific study, Professor Ferguson pleaded for the retention of the ideal of a general culture as an essential element in a sound education, and in this respect he was by no means convinced that the modern Arts curriculum is an improvement on the arrangements which preceded it. For the student of medicine, he argued, a thorough training in scientific method is essential, and it is in this work, as a teacher, that the greater part of his life had been engaged. Later in the evening, on the call of Professor John Thomson, F.R.S., the company honoured the toast of "The Chairman," and in view of the heartiness and enthusiasm with which this was received, Professor Ferguson may be well assured of the regard and confidence of his former pupils. Altogether his visit to the Club was a decided success, and no doubt the welcome he received will remain with him as an ever grateful memory.

### Consultations at Hospitals.

We observe that one of our lay contemporaries has received a letter from a "Country Doctor" urging that it should be possible for a general practitioner to send a poor patient to a hospital for the purpose merely of getting a further opinion on the case, and not in order that the patient may be retained and treated. To judge from the terms of his letter, "Country Doctor" appears to think that such a method of procedure is not possible under existing arrangements, and that the official objection to the proposal is that "it would be too much of a tax upon the time of the officials." In what isolated and remote district the writer of this letter may dwell we cannot say, but we should imagine he must be almost singular in his belief that the suggestion he advances involves a new departure. It is quite true that many modern hospital reformers have urged that out-patient departments should be restricted to the kind of work which "Country Doctor" defines in his letter. But this is not to deny that such work is already prosecuted at hospitals. As a matter of fact, indeed, it is undertaken on a large scale by every hospital boasting any measure of importance. In many such institutions not a day passes but one or more patients present themselves with an introduction from a general practitioner, and are seen by one or other member of the visiting staff. Whether the out-patient clinic should or should not be confined to this work is a question on which opinions differ. Personally we can see no difficulty in working an institution on such lines, and, indeed, there is in London one clinical service—namely, that at the Polyclinic—which is organised and conducted entirely on this principle. The future may see considerable developments in this direction. But in the meantime no advantage can arise from members of the profession announcing to the public that hospitals refrain from a form of work which, as a matter of fact, they regularly undertake.

### The Prevalence of Scarlet Fever.

During the past few days some figures have been placed before the public upon which medical practitioners may be expected to express an opinion. Our reference is to a discussion at the Metropolitan Asylums Board on the prevalence of scarlet fever, where the somewhat startling suggestion was advanced that possibly rate-supported institutions for the reception and treatment of this disease were useless in preventing its extension and spread. It seems that in 1890 there were notified in the metropolitan area 15,330 cases of scarlet fever—that is a ratio per 1,000 of population of 3.7; last year the numbers notified were 20,329, or a ratio per 1,000 of population of 4.3; and during the present year there has been a further substantial increase. Such figures appear to form an abundant justification for careful inquiry and study, with a view to discover, if possible, how more effective measures of prevention may be devised. It is certainly disappointing to find that the system of compulsory notification, and the provision of isolation hospitals at a large cost to the public, have not been followed by more conspicuous effects. The scientific position in reference to the manner of infection in scarlet fever appears at present to be a somewhat indefinite one, and there are some differences in practice related to the existing varieties of opinion on points of theory. Hence it would appear that there are good grounds for the proposal made by Professor W. R. Smith that an inquiry into the whole matter should be instituted by the Local Government Board. To be of value this ought to embrace the whole of the kingdom, as it is particularly necessary to know whether differences in regard to the criteria according to which patients are discharged from hospital do or do not coincide with a varying degree in the local prevalence of the disease. Though thus recognising that the modern policy of isolation does not seem to have been so successful as was anticipated in regard to the prevention of scarlet fever, the mortality rate shows a most gratifying improvement. In 1883 the death-rate was 12.3 per 1,000 cases, whereas in 1906 it fell to 2.9 per 1,000.

